# Matrix Metalloproteinase 8 (Collagenase 2) Induces the Expression of Interleukins 6 and 8 in Breast Cancer Cells*

**DOI:** 10.1074/jbc.M113.464230

**Published:** 2013-04-30

**Authors:** Sally Thirkettle, Julie Decock, Hugh Arnold, Caroline J. Pennington, Diane M. Jaworski, Dylan R. Edwards

**Affiliations:** From the ‡ School of Biological Sciences, University of East Anglia, Norwich Research Park, Norwich, NR4 7TJ, United Kingdom and; the § Department of Neurological Sciences, University of Vermont College of Medicine, Burlington, Vermont 05405

**Keywords:** Breast Cancer, Cancer Biology, Chemokines, Cytokine, Matrix Metalloproteinase (MMP), Neutrophil Collagenase

## Abstract

Matrix metalloproteinase 8 (MMP-8) is a tumor-suppressive protease that cleaves numerous substrates, including matrix proteins and chemokines. In particular, MMP-8 proteolytically activates IL-8 and, thereby, regulates neutrophil chemotaxis *in vivo*. We explored the effects of expression of either a WT or catalytically inactive (E198A) mutant version of MMP-8 in human breast cancer cell lines. Analysis of serum-free conditioned media from three breast cancer cell lines (MCF-7, SK-BR-3, and MDA-MB-231) expressing WT MMP-8 revealed elevated levels of IL-6 and IL-8. This increase was mirrored at the mRNA level and was dependent on MMP-8 catalytic activity. However, sustained expression of WT MMP-8 by breast cancer cells was non-permissive for long-term growth, as shown by reduced colony formation compared with cells expressing either control vector or E198A mutant MMP-8. In long-term culture of transfected MDA-MB-231 cells, expression of WT but not E198A mutant MMP-8 was lost, with IL-6 and IL-8 levels returning to base line. Rare clonal isolates of MDA-MB-231 cells expressing WT MMP-8 were generated, and these showed constitutively high levels of IL-6 and IL-8, although production of the interleukins was no longer dependent upon MMP-8 activity. These studies support a causal connection between MMP-8 activity and the IL-6/IL-8 network, with an acute response to MMP-8 involving induction of the proinflammatory mediators, which may in part serve to compensate for the deleterious effects of MMP-8 on breast cancer cell growth. This axis may be relevant to the recognized ability of MMP-8 to orchestrate the innate immune system in inflammation *in vivo*.

## Teaser

**Background:** Matrix metalloproteinase 8 is a metastasis-suppressing metalloprotease that regulates neutrophil chemotaxis in inflammation.

**Results:** MMP-8 expression by breast cancer cells is deleterious to long-term growth, but it induces IL-6 and IL-8, factors that conventionally promote malignancy.

**Conclusion:** MMP-8 influences signaling that regulates expression of proinflammatory factors.

**Significance:** These effects may contribute to how MMP-8 controls the onset and resolution of inflammation.

## Introduction

The matrix metalloproteinases (MMPs)^3^ are a family of 24 secreted proteases in humans that have fundamental roles in the catabolic turnover of extracellular matrix structures. Their activities are important in both normal and pathological tissue remodeling processes. However, the past decade has witnessed a growing appreciation of the fact that although these enzymes were originally identified by virtue of their ability to break down extracellular matrix components, they also control cell behavior via precise regulatory proteolytic cleavage of growth factors, receptors, adhesion molecules, and chemokines (1). Moreover, MMPs were originally considered to play an exclusively facilitatory role in tumor cell invasion and metastasis, but following the failure of synthetic metalloproteinase inhibitors in the clinic (2, 3), it has become recognized that MMPs and proteases of other classes can have powerful actions as suppressors of malignancy (4, 5). This is particularly the case for MMP-8, which has been reported to suppress tumor formation or metastasis, depending on the model system (6–10).

Originally identified as neutrophil collagenase, MMP-8 was the second member of the MMP family shown to cleave triple-helical collagen fibrils (11). It is found in specific granules in neutrophils but is also expressed by diverse cell types, including epithelial cells, fibroblasts, macrophages, and endothelial cells (12). Several studies have demonstrated tumor- or metastasis-suppressive activities of MMP-8. Firstly, male *Mmp8*-null animals show a dramatic increase in the incidence of carcinogen-induced skin papillomas (6). Secondly, in clonal cell lines derived from MDA-MB-435 cancer cells that display high (M-4A4) or low (NM-2C5) metastatic ability, overexpression of MMP-8 reduced the metastatic ability of M-4A4 cells, whereas ribozyme-mediated gene knockdown of MMP-8 enhanced metastasis of the NM-2C5 line (7, 13). A C/T single nucleotide polymorphism in the *MMP8* promoter that affects MMP-8 expression has been linked to survival of breast cancer patients with early-stage disease, with the high expression T allele equating to better survival (8). Higher *MMP8* RNA levels in primary breast cancers are associated with reduced lymph node metastasis and with improved relapse-free and overall survival in node-negative patients (9). The metastasis-suppressive properties of MMP-8 were confirmed in this study and shown to be dependent on its MMP activity (9). Expression of MMP-8 has also been found to be protective in human squamous cell carcinoma of the tongue and in a carcinogen-induced mouse model (10).

In addition to its suppressive roles in cancer progression, MMP-8 is protective in bleomycin-induced lung fibrosis (14), ventilator-induced lung injury (15), periodontitis (16), and allergen-induced airway inflammation (17). In contrast, *Mmp8*-deficient mice show reduced development of experimental autoimmune encephalomyelitis, suggesting that MMP-8 promotes pathology in this model of multiple sclerosis (18). It is likely that underlying all of these pathologies is the influence of MMP-8 on innate immune responses via its actions on chemokines and other immunomodulators. Cleavage of murine LPS-induced CXC chemokine (LIX) at Ser^4^-Val^5^ and Lys^79^-Arg^80^ by MMP-8 activates the chemokine, leading to enhanced neutrophil chemotaxis (19). Although other MMPs can process LIX *in vitro*, MMP-8 appears to be essential *in vivo* for LPS-stimulated neutrophil chemokinesis. MMP-8 also executes comparable processing of interleukin 8 (CXCL8/IL-8) and CXCL5/ENA-88, the human orthologues of LIX (19). Thus, MMP-8 released from neutrophils and potentially other cellular sources at sites of inflammation activates LIX/IL-8, creating a “feed-forward” response that drives further neutrophil recruitment and potentially orchestrating subsequent events in the inflammatory process, including its resolution. In the absence of MMP-8, inflammation is persistent and non-resolving, which is the situation found in the delayed skin wound healing seen in *Mmp8*-null mice (20).

On the basis of the known tumor- or metastasis-suppressive effects of MMP-8 in several cancers and its links to inflammatory responses, we sought to explore in more detail its actions on cytokine and chemokine networks elaborated by breast cancer cells. We show here that expression of catalytically active MMP-8 leads to increased production of the proinflammatory mediators IL-6 and IL-8 in multiple breast cancer cell lines but not in normal mammary epithelial cells. In turn, IL-6 enhances endogenous *MMP8* expression in breast cancer cells, and IL-8 is responsible in part for the increased expression of IL-6. Our data support the idea that MMP-8 can create a self-reinforcing protease-immunomodulatory circuit that may underpin its function in diverse physiological repair and defense mechanisms. However, we also show that expression of catalytically active MMP-8 is not sustainable in breast cancer cells in long-term culture without additional stochastic events that allow the emergence of high-expressing clones. These results may, therefore, help to integrate the roles of MMP-8 in inflammation and as a negative regulator of tumor progression.

## EXPERIMENTAL PROCEDURES

### 

#### 

##### Cell Lines and Plasmids

MDA-MB-231 and MCF-7 breast cancer cell lines (ATCC) were maintained in DMEM + GlutaMAX^TM^ (Invitrogen) supplemented with 10% fetal bovine serum. The SK-BR-3 breast cancer and G361 melanoma cell lines (ATCC) were maintained in McCoy's 5A medium (Invitrogen) supplemented with 10% fetal bovine serum.

The full-length coding sequence of human MMP-8 was cloned into the eukaryotic expression vector pcDNA4^TM^ V5-His A plasmid (Invitrogen) using the BamH I and EcoR I restriction sites. A catalytically inactive mutant (E198A) was generated using a mutagenesis PCR kit (Stratagene, La Jolla, CA) and the following primers: 5′-CTTGTTGCTGCTCAT*GCA*TTTGGCCATTCTTTGG and 5′-CCAAAGAATGGCCAAA*TGC*ATGAGCAGCAACAAG. Empty vector pcDNA4^TM^ V5-His A was used as a control.

##### Transient and Stable Transfection

To obtain transient overexpression of MMP-8, cells were plated 24 h prior to transfection at a density of 3.1 × 10^4^/cm^2^ and transfected at 80–90% confluency with 1 μg of plasmid DNA using LipoD293 (SignaGen Laboratories, Rockville, MD). Conditioned medium and RNA were collected 48 h post-transfection.

Stable overexpression of MMP-8 in MDA-MB-231 cells was achieved using calcium phosphate transfection, and selection in DMEM + GlutaMAX^TM^ supplemented with 10% fetal bovine serum and 40 μg/ml zeocin (Invitrogen). Isolated clones were generated by serial dilution of a polyclonal pool of stably transfected cells, and expression of MMP-8 was assessed by Western blot analysis and real-time RT-PCR.

##### Preparation of Cell Lysates and Conditioned Media

Cell lysates were prepared in radioimmune precipitation assay lysis buffer supplemented with protease inhibitors (Roche). Conditioned media was collected from MDA-MB-231 stably transfected cells after 24 h serum starvation or 48 h after siRNA transfection. For Western blot analysis, the conditioned media were concentrated 20× and resuspended in reducing Laemmli sample buffer.

##### Cytometric Bead Array

Serum-free conditioned media from cells transiently transfected with pcDNA4 vector control, WT MMP-8, and E198A mutant MMP-8 plasmid constructs for 48 h were used in the commercially available human inflammatory cytokine kit (BD Biosciences, catalog no. 551811) according to the guidelines of the manufacturer, and a BDFACSAria^TM^ II flow cytometer was used to determine the levels of IL-8, IL-1β, IL-10, TNF, IL-6, IL-12p70 in the conditioned media from the transiently transfected cells.

##### Western Blot Analysis

Cell lysates and 20× concentrated conditioned media samples were reduced and denatured in Laemmli sample buffer, and equal amounts of total protein were loaded on a 10% polyacrylamide gel. Protein samples were transferred onto polyvinylidene fluoride membrane (Millipore, Billerica, MA), blocked in PBS-0.1% Tween containing 5% bovine serum albumin, and incubated overnight at 4 °C with primary antibodies diluted in PBS-0.1% Tween containing 5% bovine serum albumin (MMP-8, Abcam, catalog no. ab81286 at 1:2000; V5, Invitrogen, catalog no. 460705, at 1:5000). Membranes were washed with PBS-0.1% Tween and incubated for 2 h with infrared-labeled secondary antibodies diluted in PBS-0.1% Tween with 5% bovine serum albumin (donkey anti-mouse IRDye 800CW and donkey anti-rabbit IRDye 800CW, Li-Cor Biosciences, Lincoln, NE). Finally, membranes were washed in PBS-0.1% Tween and imaged using an infrared scanner (Odyssey, Stamford, CT).

##### ELISA

Conditioned media collected after 24 h serum starvation were used to assess the total levels of IL-6, IL-8, and TNFα using specific ELISAs (eBiosciences, San Diego, CA) following the guidelines of the manufacturer.

##### Quantitative RT-PCR

Cell pellets were harvested in RNA-Bee RNA isolation reagent (Amsbio, Lake Forest, CA) followed by a combination method of chloroform extraction and RNA isolation using the SV Total RNA isolation system (Promega, Madison, WI) and stored at −80 °C.

RNA quantity and quality were assessed by *A*_260_/*A*_280_ and *A*_230_/*A*_280_ absorbance ratios using the Nanodrop (Thermo Fisher Scientific, Waltham, MA). A total of 1 μg of RNA was reverse-transcribed to 50 ng/μl cDNA, and real time RT-PCR was performed on an ABI 7700 PCR machine (Invitrogen) using the following cycles: 2 min at 50 °C and 10 min at 95 °C followed by 40 cycles of 15 s at 90 °C and 1 min at 60 °C. IL-6 and IL-8 cDNA expression was quantified using human-specific 5′FAM-3′TAMRA Taqman gene expression primer/probe sets (Hs00985639_m1 and Hs00174103_m1, Invitrogen), and PAR-2 cDNA expression was quantified using the following primers: 5′CGTCCAGTGGAGCTCTGAGT-3′ and 5′-GCAGGAGAGAGAGGCTGCTA-3′ and universal probe #3 (Roche). Expression levels were normalized to levels of 18 S rRNA (forward primer 5′-GCCGCTAGAGGTGAAATTCTTG-3′, reverse primer 5′-CATTCTTGGCAAATGCTTTCG-3′, and probe ACCGGCGCAAGACGGA). For detection of human MMP8 expression, custom-made primers (Primer Design) were used.

##### Cell Stimulation and Inhibition Experiments

Recombinant human IL-6 (R&D Systems, Minneapolis, MN, catalog no. 206-IL/CF) and IL-8 (R&D Systems, catalog no. 208-IL/CF) were added to serum-starved (2 h) MDA-MB-231 cells at 0.3, 0.6, and 100 ng of IL-6 and 0.5, 1.5, and 100 ng of IL-8 for 24 h.

For inhibitor treatments, cells were plated at 2.6 × 10^4^/cm^2^ 24 h before addition of inhibitors in serum-free conditions. Inhibitors used were as follows: p38 inhibitors: 1 μm SB203580 and 1 μm BIRB (catalog nos. CAY13067 and CAY10460, Cayman Chemicals); MKK1 inhibitors: 0.1 μm PD0325901 and 10 μm U0126 (catalog nos. CAY13034 and CAY70970, Cayman Chemicals); PI3K inhibitors: 1 μm Wortmannin and 0.5 μm PI-103 (catalog no. W1628, Sigma, and catalog no. 10009209, Cayman Chemicals); PKC inhibitor: 360 nm bisindolylmaleimide (catalog no. 203290, Calbiochem); JNK inhibitor: 20 μm SP600125 (catalog no. S5567, Sigma); NFκB inhibitor: 10 μm BAY 11-7082 (catalog no. 196871, Calbiochem); TGFβ inhibitor: SB431542 (catalog no. CAY13031, Cayman Chemicals); and MMP-8 inhibitor: 1 μm DL111 (a gift from Prof. Vincent Dive, CEA Saclay, Gif-sur-Yvette, France). Inhibitors were replenished after 24 h, and conditioned media were collected after a total incubation of 48 h.

##### SiRNA Knockdowns

MDA-MB-231 cells stably overexpressing wild-type MMP-8 were transfected at 60% confluency with 25 nm of either IL-6 (siGENOME SMARTpool M-007993-02-0005, Dharmacon, Lafayette, CO), IL-8 (siGENOME SMARTpool M-004756-00-0005, Dharmacon), or control siRNA (siGENOME non-targeting siRNA pool #1 D-001206-13-05, Dharmacon) using DharmaFECT 1 transfection reagent (Dharmacon, catalog no. T-2001-02). RNA was collected 48 h later for analysis. Cells transfected with MMP-8 siRNA (siGENOME SMARTpool M-005969-00-0005, Dharmacon) or F2RL1 (PAR-2) (siGENOME SMARTpool, M-005098-01-005, Dharmacon) were transfected a second time 24 h later. Serum-starved conditioned media and cell pellets for RNA and protein isolation were collected 48 h after the second transfection.

##### Colony Formation Assay

MDA-MB-231 cells were transfected using the calcium phosphate method for stable overexpression and selected using 200 μg/ml zeocin. Approximately 20 days after transfection, individual colonies were visualized by staining with methylene blue for 30 min at room temperature.

##### Statistical Analysis

Unless stated otherwise, data were tested using two-tailed Student's *t* tests and are presented as mean ± S.E.

## RESULTS

### 

#### 

##### MMP-8 Up-regulates of IL-6 and IL-8 Production by Breast Cancer Cells

**FIGURE 1.**
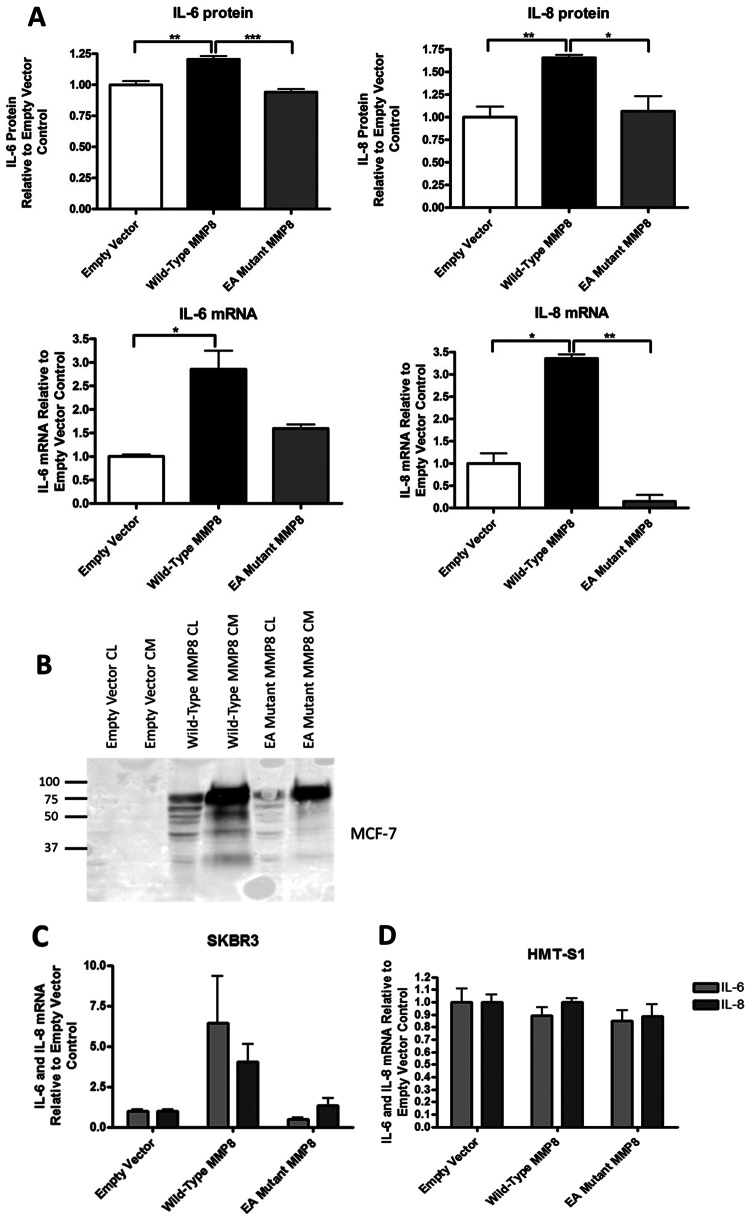
**Transient expression of WT MMP-8 causes an up-regulation of IL-6 and IL-6 protein and mRNA expression that is dependent on the catalytic activity of MMP-8.** *A*, MCF-7 cells were transiently transfected with pcDNA4 empty vector, wild-type MMP-8, and E198A mutant MMP-8 (*EA*). *Top panel*, FACS cytokine bead array measurements of IL-6 and IL-8 protein levels in serum-free conditioned media collected 24 h after transfection. *Lower panel*, RNA quantification by real-time TaqMan RT-PCR of IL-6 and IL-8 mRNAs in cells. *, *p* < 0.05; **, *p* < 0.01; ***, *p* < 0.001. *B*, Western blot analysis of WT MMP-8 and E198A MMP-8 expression in transfected MCF-7 cells. *C*, RNA quantification by real-time TaqMan RT-PCR of IL-6 and IL-8 mRNAs in SK-BR-3 cells transiently transfected with pcDNA4 empty vector, WT MMP-8, and E198A mutant MMP-8. *D*, RNA quantification by real-time TaqMan RT-PCR of IL-6 and IL-8 mRNAs in HMT-3552 S1 cells transiently transfected with pcDNA4 empty vector, WT MMP-8, and E198A mutant MMP-8. *CL,* cell lysate; *CM,* conditioned media.

To explore the effects of MMP-8 on inflammatory mediators generated by breast cancer cells, we generated a full-length wild-type human MMP-8 expression construct in pcDNA4 and a mutant in which the essential glutamic acid residue in the catalytic core motif HE^198^FGH was altered to alanine (E198A mutant MMP-8). These constructs were transiently transfected into MCF-7 cells, and after 24 h, serum-free conditioned media were analyzed by a FACS cytokine bead array for levels of IL-6, IL-8, IL-10, IL-1β, TNFα, and IL-12p70 released. Like IL-8, IL-10 is known to be a substrate of MMP-8 (14), whereas IL-1β has been shown to up-regulate MMP-8 expression (21). Expression of IL-6 and -8 in MCF-7 cells is reported to be low because of inhibition by the estrogen receptor (22–25). We detected a 20% increase in IL-6 and a 65% increase in IL-8 protein levels in the conditioned media in WT-MMP-8-expressing cells compared with empty vector (EV) (Fig. 1*A*, *upper panels*). This increase was dependent on the catalytic activity of MMP-8 because cells transfected with the E198A mutant MMP-8 showed levels similar to the control cells. None of the other inflammatory mediators were detected in the MCF-7 cell-conditioned media at measurable levels. Wild-type and E198A mutant MMP-8 were expressed and released into the conditioned media at equivalent levels as determined by Western blotting (Fig. 1*B*). We anticipated that the increased detection of the inflammatory mediators might be attributable to release of matrix- or cell-associated IL-6 and IL-8 by MMP-8. However, the increases in IL-6 and IL-8 protein were mirrored at the mRNA level, with WT MMP-8 increasing both IL-6 and IL-8 mRNA levels 2- to 3-fold. Similar results were obtained with another breast cancer cell line (SK-BR-3) transiently transfected with WT MMP-8 and E198A MMP-8 (Fig. 1*C*), but this phenomenon was not seen on transient transfection into a non-cancerous breast epithelial cell line, HMT-3522 S1 (*D*).

**FIGURE 2.**
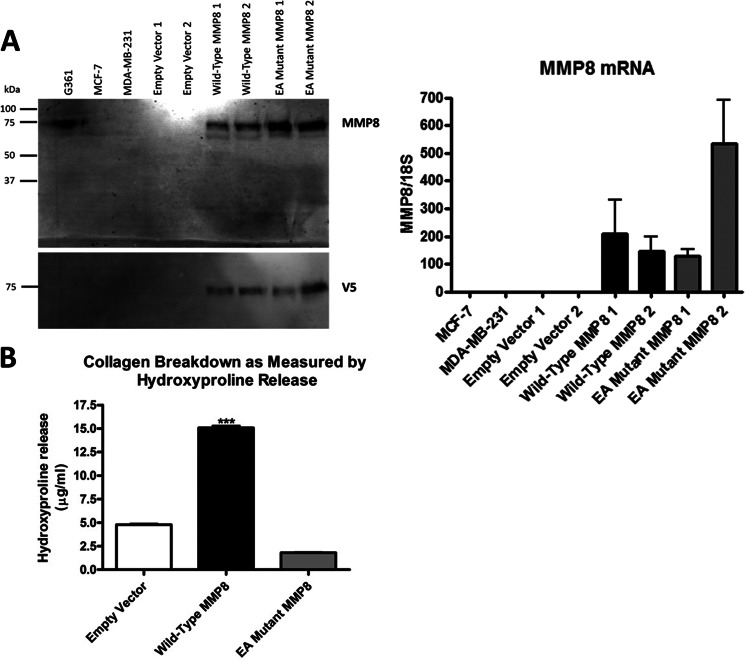
**Generation of an i*n vitro* model to examine the effects of MMP-8 expression in MDA-MB-231 breast carcinoma cells.** *A*, expression of MMP-8 in serum-free conditioned media of MDA-MB-231 cells stably transfected with pcDNA4 empty vector, wild-type MMP-8, and E198A mutant MMP-8 as shown by Western blot analysis using both anti-MMP8 antibody (*top panel*) and anti-V5 antibody (*bottom panel*). Media from G361 melanoma cells were included as a positive control for endogenous MMP-8 and parental MCF-7 and MDA-MB-231 cell conditioned media as negative controls. All media were concentrated 20×. *Right panel,* RNA quantification by real-time TaqMan RT-PCR of MMP-8 mRNAs in the same cells. *B*, collagen-degrading activity of MDA-MB-231 cells stably transfected with pcDNA4 empty vector, WT MMP-8, and E198A mutant MMP-8 cultured within collagen gels and measured indirectly by hydroxyproline release. ***, *p* < 0.001.

**FIGURE 3.**
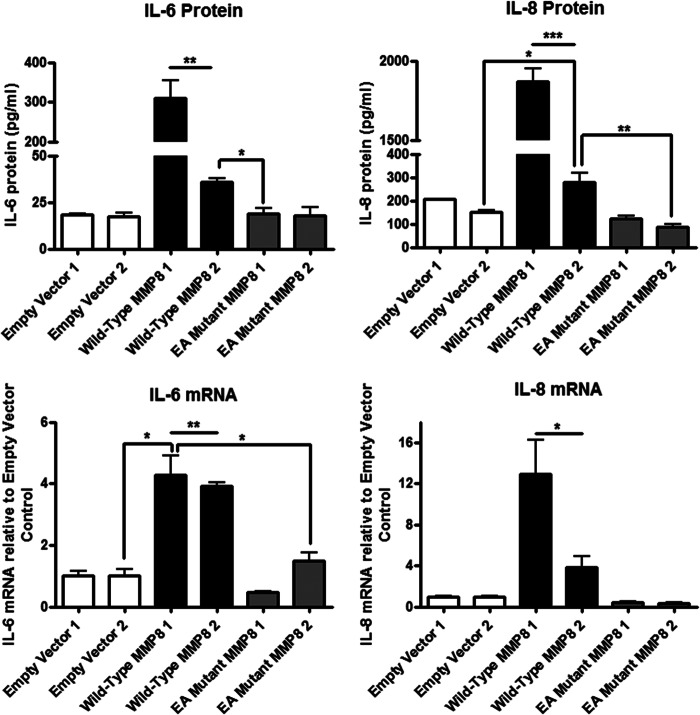
**Long-term stable MMP-8 overexpression also causes an up-regulation of IL-6 and IL-6 protein and mRNA expression that is dependent on the catalytic activity of MMP-8.** Two independent clones of MDA-MB-231 cells stably transfected with pcDNA4 empty vector, wild-type MMP-8, and E198A mutant MMP-8 were analyzed. *Top panel*, ELISA measurements of IL-6 and IL-8 protein levels in serum-free conditioned media collected 24 h after serum starvation. *Lower panel*, RNA quantification by real-time TaqMan RT-PCR of IL-6 and IL-8 mRNAs in the same cells. Both wild-type MMP-8 clones 1 and 2 were different compared with all other cells by at least *p* < 0.05 or *p* < 0.01 unless shown otherwise *, *p* < 0.05; **, *p* < 0.01; ***, *p* < 0.001. *EA,* E198A mutant MMP-8.

The human breast cancer cell lines we examined (*MDA-MB-231*, *MCF7*, and *SK-BR-3*; Fig. 2*A* and data not shown) did not express MMP-8 at a level that was detectable by Western blot analysis, and only MDA-MB-231 cells expressed a very small amount of endogenous *MMP8* that was detectable by TaqMan quantitative real-time RT-PCR (data not shown, see also Fig. 5*C*). Using the highly metastatic MDA-MB-231 cells (estrogen receptor-/progesterone receptor-/HER2), we generated stably transfected clones carrying the WT MMP-8, E198A mutant, or EV control vector. Fig. 2*A* shows that comparable levels of the wild-type and catalytically inactive MMP-8 proteins were expressed and released into the conditioned media and that *MMP8* transcript levels originating from the transfected genes were also similar. The levels of MMP-8 protein expression achieved in these transfected clonal lines were similar to that of endogenous MMP-8 in conditioned media from the G361 melanoma cell line, which was included as a control. The major MMP-8 band was 75 kDa, likely corresponding to pro-MMP-8, but a faster migrating band was detected by the MMP-8 antibody, although this could be a C-terminally processed form because it was undetectable by the antibody toward the V5 tag. However, functional collagenolytic activity of the wild-type MMP-8 was confirmed by demonstration of elevated hydroxyproline release compared with the E198A mutant-expressing cells and the empty vector control (Fig. 2*B*). Analysis of IL-6 and 8 protein production by ELISA of conditioned media from two independent clonal isolates of WT MMP-8 stably transfected MDA-MB-231 cells (Fig. 3, *upper panels*) showed a 2- to 12-fold increase in IL-6 and an 2- to 8.5-fold increase in IL-8 protein in comparison to EV control cells. Again, the E198A inactive mutant MMP-8-expressing cells showed no increases compared with the EV control cells. Also, as with the transiently transfected MCF-7 cells, we found that in the MDA-MB-231 stably transfected cells, mRNA levels were increased 4-fold for IL-6 and 4- to 12-fold for IL-8 in stable WT MMP-8-expressing MDA-MB-231 clones (Fig. 3, *lower panels*), mirroring the increased levels of the soluble proteins.

We conclude that the effect of MMP-8 on the inflammatory mediators is primarily at the level of gene expression and that its catalytic activity is required because all cells overexpressing the E198A mutant show IL-6 and IL-8 protein and mRNA levels comparable with those of control-transfected cells. Furthermore, the induction of IL-6 and IL-8 was observed for all breast cancer cell lines examined (MCF-7, SK-BR-3, and MDA-MB-231) but not for HMT-3522 S1 normal mammary epithelial cells.

##### MMP-8 Expression Is Detrimental to Breast Cancer Cell Growth

**FIGURE 4.**
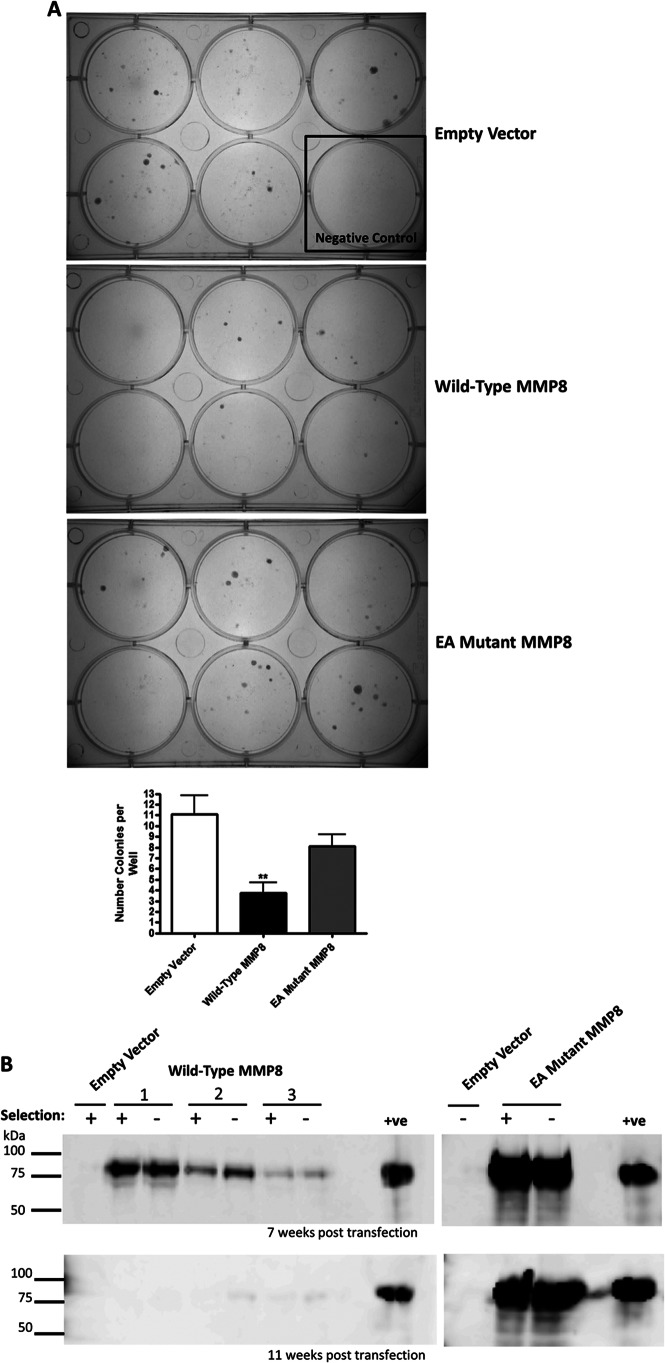
**Wild-type MMP-8 reduces colony formation of MDA-MB-231 cells, and expression is shut down by cells.** *A*, colonies visible by 20 days post-transfection stained with methylene blue. The control well consisted of cells that were untransfected and so died under zeocin selection as a control for cell death. *EA*, E198A mutant. *B*, Western blot analysis using an anti-MMP-8 antibody on 20× concentrated serum-free conditioned media, tracking the loss of expression of wild-type MMP-8 from stably transfected MDA-MB-231 cells. Three pools of wild-type MMP-8 stables (*1–3*), were cultured alongside a pool of cells transfected with the control vector, and E198A mutant MMP-8 were maintained with (+) or without (-) zeocin selection. *Top panel*, cells 7 weeks post-transfection. *Bottom panel*, cells 11 weeks post-transfection. The positive control used was conditioned media from HEK-293T cells transiently transfected with WT MMP-8. *, *p* < 0.05; **, *p* < 0.01; ***, *p* < 0.001.

When MDA-MB-231 cells were transfected with our suite of expression vectors and placed under antibiotic (zeocin) selection, after 20 days we observed a 50–60% reduction in colony formation by cells expressing WT MMP-8 compared with EV or E198A mutant MMP-8 (Fig. 4*A*). However, in shorter-term experiments following transient transfection of either WT or E198A mutant MMP-8 into MCF-7 or MDA-MB-231 cells for up to 72 h, we saw no effects on cell growth or apoptosis (data not shown). We therefore monitored independent pools of transfected MDA-MB-231 cells carried forward for up to 3 months in culture with or without zeocin selection (Fig. 4*B*). Although expression was initially comparable early after transfection (data not shown), by 7 weeks, levels of WT MMP-8 were reduced compared with E198A mutant MMP-8 (Fig. 4*B*, *upper panel*). By 11 weeks in culture, WT MMP-8 expression was barely detectable in any of the cell pools regardless of zeocin selection, whereas expression of E198A mutant MMP-8 remained at a consistently high level, again regardless of selection (Fig. 4*B*, *lower panel*). PCR analysis of genomic DNA isolated from the cell populations from this experiment confirmed that the transfected *MMP8* expression vectors were present and at the same levels in both WT and E198 mutant MMP-8 transfected cells after 11 weeks (data not shown), suggesting that the WT *MMP8* expression cassette was silenced during continued passage of the cells. Moreover, IL-6 and IL-8 expression returned to base line in the cell pools that lost MMP-8 expression (data not shown).

These data, therefore, support a causal link between MMP-8 and up-regulation of the expression and secretion of IL-6 and IL-8. Furthermore, they argue that the occasional stably transfected clones that maintain WT MMP-8 expression long-term, for longer than 3 months, such as those shown in Fig. 3, have likely undergone phenotypic alteration to compensate for the detrimental effects of MMP-8. This conclusion was supported by the observations that neither knockdown of MMP-8 in the “long-term” WT MMP-8-expressing cells or blockade of its activity using a selective small molecular weight inhibitor, KL-111, led to a reduction in IL-6 or IL-8 production (data not shown). These data emphasize that additional compensatory mechanisms that are independent of MMP-8 are operational to allow breast cancer cells to maintain elevated expression of the otherwise deleterious MMP.

##### MMP8, IL-6, and IL-8 Are Components of a Self-reinforcing Loop

**FIGURE 5.**
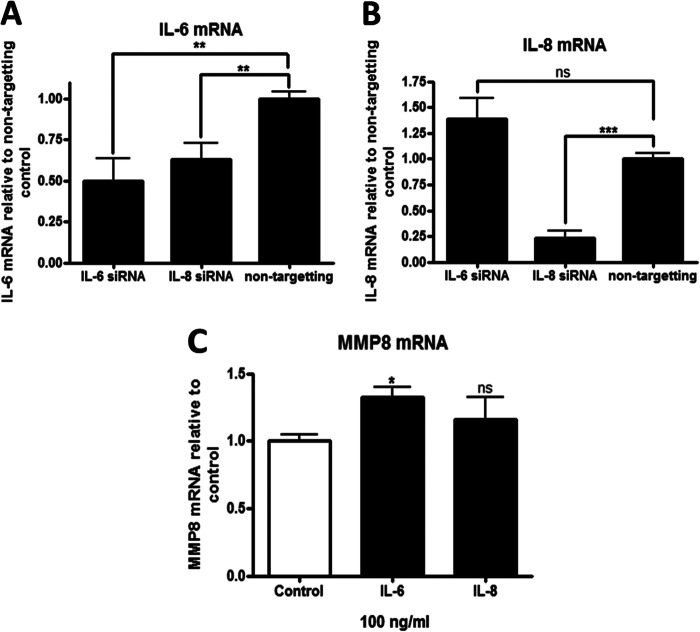
**MMP-8, IL-6, and IL-8 exist as part of an interconnected regulatory circuit.** *A*, RNA quantification by real-time TaqMan RT-PCR of IL-6 mRNA levels in WT MMP-8 overexpressing MDA-MB-231 cells after siRNA knockdown of IL-6 or IL-8 for 48 h. *B*, RNA quantification by real-time TaqMan RT-PCR of IL-8 mRNA levels in WT MMP-8 overexpressing MDA-MB-231 cells after siRNA knockdown of IL-6 or IL-8 for 48 h. *C*, RNA quantification by real-time TaqMan RT-PCR of endogenous MMP-8 in naïve, non-transfected MDA-MB-231 cells treated with recombinant human IL-6 and IL-8 for 24 h. *, *p* < 0.05; **, *p* < 0.01;***, *p* < 0.001; *ns*, not significant.

Because IL-6 and IL-8 are powerful proinflammatory mediators that exert effects on the expression of many genes, we sought to determine whether their up-regulation might link them into a dynamic self-regulating network. To determine whether a feedback loop is operating in the long-term WT MMP-8-expressing MDA-MB-231 cells, the cells were treated with siRNAs to IL-6 and IL-8 and levels of both cytokines measured by TaqMan qRT-PCR. SiRNA-mediated knockdown of IL-8 expression led to a concomitant reduction of IL-6 mRNA levels by ∼40% compared with the non-targeting control (Fig. 5*A*). In contrast, knockdown of IL-6 had no effect on IL-8 mRNA expression compared with the non-targeting control siRNA (Fig. 5*B*). In separate experiments, human recombinant IL-6 and IL-8 were added to parental MDA-MB-231 cells for 24 h, and the expression level of *MMP8* determined by RT-PCR. Addition of IL-8 alone did not affect expression of *IL6* (data not shown) or *MMP8* mRNAs, but recombinant IL-6 induced endogenous *MMP8* expression by ∼20% (Fig. 5*C*). Taken together, these data suggest that MMP-8, IL-6, and IL-8 may constitute a self-reinforcing loop. MMP-8 can induce expression of IL-6 and IL-8 in short-term exposure to the protease, and IL-8 reinforces IL-6 expression, whereas the induced IL-6 may augment *MMP8* expression. In the long-term WT MMP-8 clones, where IL-6 and IL-8 production is no longer dependent upon MMP-8, IL-8 may help to sustain elevated IL-6 expression.

##### IL-6 and IL-8 Expression in Long-term MMP-8-expressing Cells Is Dependent on NF-κB Signaling and PAR2 Is Linked to IL-6 Expression

**FIGURE 6.**
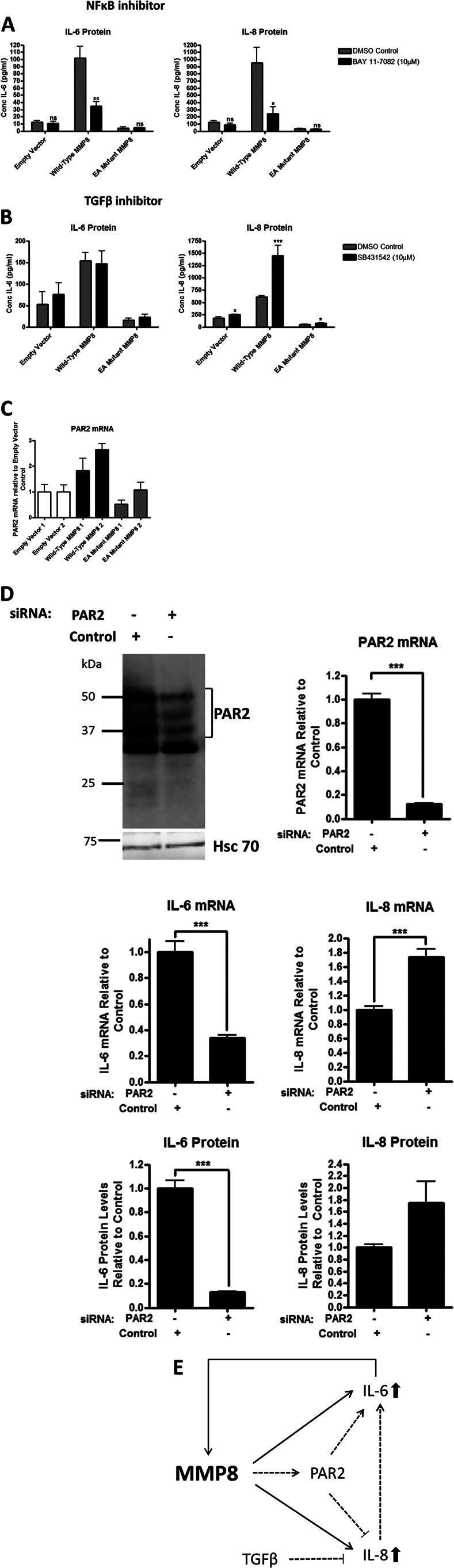
**Both IL-6 and IL-8 up-regulation occurs via NF-κB, but IL-8 is also negatively regulated by TGFβ and PAR-2.** *A*, ELISA measurements of IL-6 and IL-8 protein levels after incubation of MDA-MB-231 clonal isolates stably transfected with pcDNA4 empty vector, wild-type MMP-8, and E198A mutant (*EA*) MMP-8 after incubation with 10 μm BAY 11-7082, an NF-κB inhibitor, for 48 h. *DMSO*, dimethyl sulfoxide. *B*, ELISA measurements of IL-6 and IL-8 protein levels after incubation of MDA-MB-231 clonal isolates stably transfected with pcDNA4 empty vector, wild-type MMP-8, and E198A mutant MMP-8 after incubation with 10 μm SB431542, a TGFβR1 inhibitor, for 48 h. *C*, RNA quantification by real-time TaqMan RT-PCR of PAR-2 in MDA-MB-231 clonal isolates stably transfected with pcDNA4 empty vector, wild-type MMP-8, and E198A mutant MMP-8. *D*, *top panel*, Western blot analysis and RNA quantification by real-time TaqMan RT-PCR of PAR2 to confirm the knockdown of PAR-2 after siRNA knockdown for 48 h in wild-type MMP-8 overexpressing MDA-MB-231 clonal isolate 1. *Center panel*, RNA quantification by real-time TaqMan RT-PCR of IL-6 and IL-8 in cells after PAR-2 siRNA knockdown. *Bottom panel*, ELISA measurement on serum-free conditioned media of IL-6 and IL-8 protein levels after PAR-2 siRNA knockdown. *, *p* < 0.05; **, *p* < 0.01, ***, *p* < 0.001; *ns*, not significant. *E*, schematic showing the relationships between MMP-8, IL-6, and IL-8 in a self-reinforcing loop and pathways that may contribute to this system in breast cancer cells. On acute exposure to catalytically active WT MMP-8, breast cancer cells up-regulate the expression of IL-6 and IL-8 (*black lines* and *arrows*). IL-6 is also able to induce MMP-8 expression. *Red lines* and *arrows* show the pathways that are evident in breast cancer cells that adapt to long-term expression of WT MMP-8. Not shown on the diagram is that IL-6 and IL-8 expression is also dependent on NF-κB signaling, which may be activated by PAR-2 as well as other signaling pathways, including autocrine loops resulting from IL-6 and IL-8 induction.

We explored the effects of pharmacological inhibition of major cell signaling pathways to determine the relevance of the particular pathways to the increased expression of IL-6 and IL-8 in the long-term WT MMP-8-expressing MDA-MB-231 cells. The inhibitors used and their target pathways were as follows: PD0325901 and U0126 (MAPK-Erk pathway), SB203580 (p38), wortmannin and PI-103 (PI-3K), bisindolylmaleimide 1 (PKC), SP600125 (JNK), BAY 11-7082 (NF-κB), and SB431542 (TGFβR1). Of the inhibitors examined, only the NF-κB inhibitor BAY 11-7082 reduced secretion of both IL-6 and IL-8 by 60% compared with the vehicle control in the cells overexpressing wild-type MMP-8 (Fig. 6*A*). The TGFβR1 inhibitor SB431542 had no effect on MMP-8-induced IL-6 levels, but IL-8 levels were increased by 100% in the WT MMP-8 overexpressing cells, and the control EV and E198A mutant MMP-8-expressing cells also showed a slight induction, showing that TGFβ signaling is inhibitory to IL-8 production in MDA-MB-231 cells (Fig. 6*B*).

We also considered the possible involvement of protease-activated receptor (PAR) signaling because MMP-1 is known to cleave and activate PAR-1 (26–28). However, PAR-1 was not expressed by the parental MDA-MB-231 cells or the WT MMP-8-expressing clones (data not shown). The G protein-coupled receptor PAR-2 was considered because it is expressed by epithelial cells (29) and neutrophils (30), has a role in neutrophil migration (31), and has reported involvement in IL-6 and IL-8 up-regulation and secretion (31–33). We observed that PAR-2 expression was consistently up-regulated in the long-term WT MMP8-expressing MDA-MB-231 cells (Fig. 6*C*). Knockdown of PAR-2 by siRNA reduced IL-6 protein and mRNA expression by 90 and 60%, respectively, in the WT MMP-8 expressing cells but had the reverse effect on IL-8 expression, causing an approximate 80% increase in both RNA and protein levels (Fig. 6*D*). The schematic in Fig. 6*E* summarizes the relationships between MMP-8, IL-6, and IL-8 under acute and long-term exposure of breast cancer cells to catalytically active MMP-8 and to PAR-2 and TGF-β signaling.

## DISCUSSION

Matrix metalloproteinase 8 was the first member of the MMP family that was revealed to be tumor- or metastasis-suppressive *in vivo* (6, 7, 13). Subsequently, MMP-8 expression was found to be a marker of a lower incidence of lymph node metastasis and, consequently, to confer a better prognosis in human breast carcinoma (9). It has also been found to be protective in cancer of the tongue (10). In human melanoma, function-inactivating mutations in *MMP8* have been found at high frequency, and wild-type but not mutant forms suppressed tumor formation in soft agar assays, supporting its role as a tumor suppressor (34). This study extends these findings using a series of human mammary carcinoma cell lines, where we demonstrate that transient expression of catalytically active WT MMP-8, but not an inactive E198A mutant form, induced the expression of IL-6 and IL-8. However, sustained expression of WT MMP-8 was deleterious to breast cancer cell growth in colony formation assays, and, upon extended culture, the exogenous *MMP8* expression cassette was silenced in most cell pools, accompanied by a coordinate reduction in IL-6 and IL-8 expression. In a few instances, stable WT MMP-8-expressing clones of MDA-MB-231 cells were obtained, and these retained elevated IL-6 and IL-8 production, although we infer that additional genetic or epigenetic changes had likely occurred in these cells because their IL-6/IL-8 expression levels were no longer dependent upon MMP-8. Instead, increased IL-6 levels may in part be due to the action of IL-8 acting in an autocrine fashion and also to PAR-2, which was found to be up-regulated in the long-term WT MMP-8-expressing cells.

We detected increased levels of IL-6 and IL-8 proteins by ELISA in conditioned media of MCF-7, MDA-MB-231, and SK-BR-3 breast carcinoma cells transfected transiently with WT MMP-8, indicating that the response is likely a general one and not restricted to cancer cells that have a particular estrogen receptor status or amplified HER2. The effects on IL-6 and IL-8 were specific because we saw no increases in several other inflammatory mediators, including TNFα, IL1β, IL-10, and IL-12p70. It is known that MMP-8 carries out N-terminal processing of IL-8 and its murine ortholog LIX, leading to activation of the chemokines and increasing their neutrophil chemoattractant activity *in vivo* (19). We considered that MMP-8 might thus have led to increased detection of IL-6 and IL-8 proteins in cell-conditioned media through proteolytic cleavage of precursor or cell-associated forms of these molecules. We cannot rule this out as a contributory factor, but because the increased protein levels were reflected in increased IL6 and IL-8 mRNA abundance in WT MMP-8-transfected cells, we conclude that the principal effect of the protease is at the level of induction of gene transcription. A normal immortalized mammary epithelial cell line (HMT-3522 S1) showed no effect of WT or E198A mutant MMP-8 on the production of the interleukins, indicating that malignant transformation is a prerequisite for induction.

Interleukins 6 and 8 are well established as tumor-promoting, proinflammatory factors in numerous cancer scenarios (35, 36). Interleukin 6 acts via the membrane-bound or soluble IL-6 receptor, which binds the cytokine and then interacts with the gp130 signaling receptor, leading to activation of downstream pathways, including the JAK-1-STAT-3 pathway, MAP kinases, and NF-κB (37). Activation of IL-6 secretion, for instance via HER2 amplification, creates an autocrine signaling loop involving JAK-1/STAT-3-mediated IL-6 expression (38). Also, Src-mediated transformation of mammary epithelial cells has been shown to involve IL-6 acting in a positive feedback loop via activation of NF-κB (39). The IL-8 chemokine activates multiple pathways following its engagement with two G protein receptors (CXCR1 and CXCR2), leading to the promotion of the proliferation, survival, and migration of cancer cells (36). Recently, both IL-6 and IL-8 have also been shown to regulate breast cancer stem cell renewal (40, 41). Therefore, it appeared paradoxical to us that MMP-8, on the basis of its history as an antitumorigenic protease, would elicit the production of factors that have profoundly protumorigenic actions. Two points are critical for consideration, however, the first being the impact of MMP-8 on innate immunity and the role of the innate immune response in tumor progression and the second being the distinction between early, acute effects of catalytically active MMP-8 upon breast cancer cells and how cells cope with sustained production of the protease.

Neutrophils are the first wave of innate immune cells that are recruited to sites of acute inflammation. These triggered neutrophils release and autoactivate pro-MMP-8 from the specific granules, which then acts upon LIX (mouse) and IL-8 (human), enhancing further neutrophil chemotaxis in a feed-forward mechanism (19). The systemic absence of MMP-8 in *Mmp8*^−/−^ mice has profound effects on inflammation, the outcomes of which depend on the tissue site and the nature of the inflammation. In carcinogen-induced skin tumors or in skin wounds there is a delay in neutrophil infiltration at early times in *Mmp8*-null *versus* wild-type mice, but this eventually leads to a persistent accumulation at later time points, which exacerbates tumor development (6) and impairs wound repair (20). This is also evident in the more severe alveolar bone loss in *Mmp8*^−/−^ mice compared with the wild type in *Porphyromonas gingivalis-*induced periodontitis (16, 42) and in LPS-induced corneal inflammation (43). In all of these situations, MMP-8 is required to promote both the initial onset and the subsequent clearance of the inflammatory neutrophilic response. In some tissues, such as the lung and the liver, the absence of MMP-8 and the attendant initial reduction in neutrophil influx and reduced chemokine activation can be protective, as has been observed in models of ventilator-induced injury in the lung (15), bleomycin-induced lung fibrosis (14), and TNFα-induced lethal hepatitis (44). All of these situations are accompanied by alteration of the levels of numerous chemokines and cytokines, including reduced levels of LIX (15, 42, 44). Thus, the induction of IL-6 and IL-8 expression attributable to the catalytic activity of MMP-8 in breast cancer cells is consistent with a role for MMP-8 in the initial triggering of an acute inflammatory response. Within the microenvironments of emerging tumors, this activation may set in motion subsequent macrophage-dependent and adaptive immune system responses that act to resolve inflammation and suppress tumor progression (45).

One of the most important points to emerge from this study is that sustained, elevated expression of MMP-8 by breast cancer cells eventually impaired their growth. This did not occur as an acute response to WT MMP-8 because we saw no appreciable effects on cell growth or apoptosis over the 3–4 days during which cells were expressing the proteins following transient transfection (although subtle effects may be hard to detect in transient assays). Instead, the growth-suppressive effect of WT MMP-8 was apparent in longer-term colony formation assays. Palavalli and colleagues (34) also saw no effect on short-term growth of cultured Mel-STR melanocytes following expression of human WT MMP-8, but there was substantial inhibition of colony growth in soft agar. These effects on growth were not apparent in an earlier study that had transfected a mouse *Mmp8* expression vector into murine B16-F10 cells, so it is possible that the growth effects may be species-specific (9). In our experiments, serial passage of transfected breast MDA-MB-231 carcinoma cells expressing WT MMP-8 led, over time, to a progressive loss of expression of the protease. This was not observed for cells expressing the E198A catalytically dead mutant, indicating that it was a result of MMP-8 protease activity. The suppression occurred even when the cells were maintained under continuously enforced zeocin selection. We confirmed that the full *MMP8* expression cassette was retained in these cells, indicating that expression was likely lost through epigenetic silencing. A comprehensive analysis of the transcriptional status of the entire *MMP* family in relation to their epigenetic parameters (DNA methylation, histone modifications, and microRNAs) identified *MMP8* as displaying strict epigenetic silencing in multiple cancer cell lines, consistent with its antitumorigenic role (46). In our long-term MDA-MB-231 transfected cell cultures, loss of expression of WT MMP-8 coincided with a return to base-line levels for the expression of IL-6 and IL-8, which further supports a causal connection between MMP-8 and the elevated expression of the inflammatory mediators.

In the rare MDA-MB-231 clones that gave sustained elevated expression of WT MMP-8, although IL-6 and IL-8 levels were also maintained, the expression of the interleukins was no longer dependent on the presence of MMP-8 because neither siRNA-mediated knockdown of MMP-8 nor treatment with DL-111, an MMP-8-selective inhibitor, attenuated their expression. We infer from these data that IL-6 and IL-8 may act to overcome the growth-suppressive effects of WT MMP-8. However, these factors possibly act together with other growth-regulatory factors whose expression is sensitive to WT MMP-8 because their combined knockdown did not decrease cell growth in short-term assays (data not shown). We do not yet know the identities of the molecules that confer the continued ability of breast cancer cells to grow while expressing catalytically active MMP-8. It is clear from our knockdown experiments that IL-8 is, at least in part, responsible for the sustained expression of IL-6. Data from other groups support an autocrine feedback loop for IL-6 in breast cancer cells involving NF-κB signaling linked to control of IL-6 expression through the microRNAs let-7 (39) and miR-200c (47). Consistent with this we see a dependence of both IL-6 and IL-8 production on NF-κB signaling. We therefore speculate that IL-6 and IL-8 create a self-reinforcing loop that allows the MDA-MB-231 cells to continue growth independently of the inhibitory action of MMP-8.

It has been observed previously that the delayed wound healing seen in *Mmp8*^−/−^ mice correlated with reduced levels of active TGF-β1 (20). Altered TGFβ signaling could contribute to the WT MMP-8-mediated up-regulation of IL-8 expression we have observed because IL-8 levels were enhanced in MDA-MB-231 cells by pharmacological inhibition of TGFβ signaling. Another potential contributory mechanism for the sustained growth of WT MMP-8-expressing MDA-MB-231 cells may be the increased expression of PAR-2 that we observed in these cells compared with cells expressing the E198A mutant MMP-8. Protease-activated receptor 2 is a widely expressed member of the PAR family of G-protein-coupled receptors that is known to be activated by several serine proteases, including trypsin and matriptase, leading to activation of NF-κB signaling (48, 49). Knockdown of PAR-2 expression in the WT MMP-8-expressing cells led to a reduction of IL-6 production, but, in contrast, this led to a further augmentation of IL-8 expression. Although MMP-1 (collagenase 1) is known to cleave and activate PAR-1 (26–28), we have no evidence to suggest that MMP-8 is acting directly through PAR-2. Indeed, as discussed above, in the long-term WT MMP-8-expressing clones, IL-6/IL-8 are decoupled from MMP-8 action. It is possible, however, that among the genetic/epigenetic changes during clonal evolution that have allowed MDA-MB-231 cells to tolerate WT MMP-8 may be the increased production of proteases that act through PAR-2. However, this likely contributes only to the IL-6 expression status because PAR-2 negatively regulates IL-8 expression (Fig. 6*E*).

In conclusion, therefore, our experiments reveal a novel action of catalytically active MMP-8 in breast cancer cells that results in increased production of key regulators of tumor growth and inflammation, IL-6, and IL-8. These effects occur upon acute expression of the enzyme and are not apparent in non-transformed mammary epithelial cells, suggesting that they may be linked with tumor progression. Although the identities of the MMP-8 substrates that are the direct mediators of its effects on growth and the expression of the inflammatory mediators remain to be firmly established, our data implicate the involvement of both TGFβ- and PAR-2-mediated signaling. We suggest that the induction of proinflammatory mediators such as IL-6 and IL-8 is relevant to the mechanisms by which MMP-8 orchestrates the onset and resolution of inflammation *in vivo* in tumors and during tissue repair.

## Footnotes

3 The abbreviations used are:

MMP: matrix metalloproteinase
LIX: LPS-induced CXC
EV: empty vector
PAR: protease-activated receptor.
